# Advancing Real-World Evidence Generation: Growth and Lessons Learned from the Early Years of Cosmos

**DOI:** 10.1055/a-2842-3407

**Published:** 2026-04-24

**Authors:** Meghan Howat, Steven Martell, Yasir Tarabichi, David C. Kaelber, Selina S. Chen, Harry Freedman

**Affiliations:** 1 Epic Systems Corporation, Verona, Wisconsin, United States; 2 Center for Clinical Informatics Research and Education, The MetroHealth System, Cleveland, Ohio, United States; 3 Departments of Internal Medicine, Pediatrics, and Population and Quantitative Health Sciences, Case Western Reserve University, Cleveland, Ohio, United States; 4 John A. Burns School of Medicine, Hawai'i Pacific Health and the Department of Pediatrics, Honolulu, Hawaii, United States

**Keywords:** big data, real world data, real world evidence, electronic health records and systems, informatics, research database, clinical data repository, health data

## Abstract

**Objective:**

Cosmos is a vendor-facilitated platform created in collaboration with healthcare systems using electronic health records (EHRs) from Epic Systems Corporation. Participating organizations can use the platform to advance research and apply insights at the point of care. This manuscript outlines evolutions in the platform's infrastructure, growth of available data, and usage.

**Methods:**

Data from participating systems is sent via encrypted Health Level Seven Clinical Document Architecture to the Cosmos host at Epic Systems Corporation. Deduplicated data are stored in two relational Structured Query Language (SQL) databases, which can be accessed remotely. Aggregate counts from a limited dataset (LDS) are accessed through a no-code data visualization and analysis tool. Line-level data from an expert-determined de-identified dataset are accessed in a secure virtual computing environment. Data quality and user support frameworks facilitate a feedback loop improving data quality and researcher efficiency. Platform growth was measured as participating organization counts, data elements, and proportion of patient records with data spanning over 5 years. Medical literature was reviewed for peer-reviewed studies using Cosmos data.

**Results:**

Over 330 organizations across Canada, Lebanon, Saudi Arabia, and the United States submit data to Cosmos. The platform includes more than 300 million deduplicated patient records, 39% of which have over 5 years of clinical data. Ongoing expansion of data elements within Cosmos has allowed the platform to serve as the data source for over 100 peer-reviewed studies across multiple disciplines.

**Discussion:**

With its expanding size, tooling, and breadth of data elements, Cosmos may enable a wide range of study types. Cosmos remains subject to limitations such as variation in workflows, documentation error, and bias inherent to a population sourced from healthcare organizations using a single EHR vendor.

**Conclusion:**

Cosmos demonstrates a use case of vendor-facilitated collaboration to enable a broad spectrum of research.

## Introduction

Cosmos ( *https://cosmos.epic.com* ) is a real-world healthcare research platform containing anonymized electronic health record (EHR) data managed by Epic Systems Corporation. Cosmos is part of a trend toward multi-institutional health data repositories facilitating observational research including networks with a central coordinating health system, 1 2 3 4 health-system-led collaborations, 5 6 and vendor-based initiatives. 7 8 9 These platforms leverage EHR adoptions and health information exchange networks to securely collect data across sites while maintaining patient privacy. 9 10 Despite challenges in standardizing data and safeguarding patient privacy while optimizing research utility, they have proven capable of enabling tools for clinical decision-making and conducting research beyond what a single institution's EHR system can provide. 11

De-identified data in Cosmos are stored centrally in Verona, Wisconsin, United States with safeguards against patient re-identification meeting Standard for Attestation for Federated EHR (SAFE) standards 12 13 and Health Insurance Portability and Accountability Act (HIPAA) guidelines for limited 14 15 and de-identified datasets. 16 17 At the time of this manuscript's submission (February 2026), the platform aggregates data from four countries: Canada, Lebanon, Saudi Arabia, and the U.S. (including all 50 states and Washington, D.C.). After 7 years organizations began submitting data, this includes over 300 million linked patient records with data deduplicated between organizations from inpatient, outpatient, and surgical settings in community and academic medical centers, federally qualified health centers, critical access hospitals, specialty practices, and other healthcare-delivery organizations. 18 19 Peer-reviewed studies using data from Cosmos have analyzed rare populations at a novel scale, 20 addressed contemporary medical questions, 21 22 or applied of novel data elements. 23 24 In this manuscript we describe updates to the platform's infrastructure, governance, research applications, and limitations and opportunities since Tarabichi et al 25 first described the platform in 2021.

## Methods

### Infrastructure and Maintenance

#### Data Submission

**Fig. 1:**
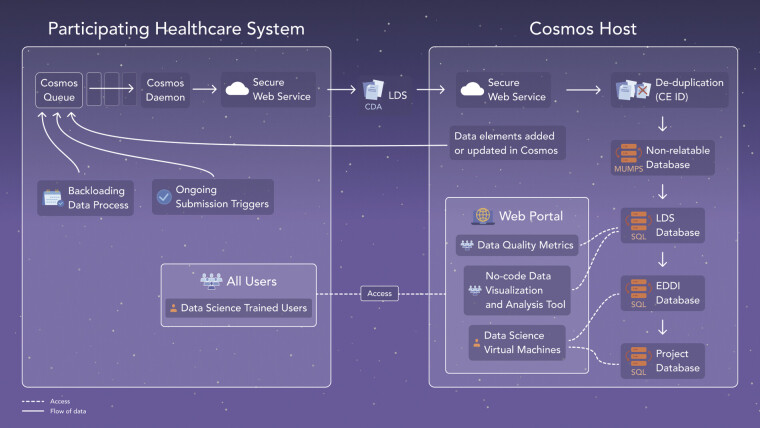
Current schematic for the Cosmos architecture. The backloading process, ongoing submission triggers, and added or updated data elements in the Cosmos database cause data to move onto the Cosmos queue within the participating healthcare system's EHR. The Cosmos daemon triggers submission of data elements permitted as part of a limited dataset (LDS) from this queue to the Cosmos host via encrypted HL7 C-CDA documents over the Epic Care Everywhere network. All participating healthcare systems communicate with the same Cosmos host. Patient deduplication is performed by using a hashed copy of a Care Everywhere ID, after which data are filed in a Massachusetts General Hospital Utility Multi-Programming System (MUMPS) non-relational database, and then a Structured Query Language (SQL) relational LDS database. Transforms on a copy of the LDS database file data into an expert determination de-identified (EDDI) SQL database. Data from the EDDI database may be filed into project-specific SQL databases. All users can access data on a web portal using a no-code data visualization and analysis tool for the LDS, and data-science-trained users can additionally access data using a Data Science Virtual Machine for the EDDI and project-specific databases. Source: © 2026 Epic Systems Corporation.

Before submitting data, Cosmos requires mapping of data based on Unified Medical Language System (UMLS) 26 ontologies, such as International Classification of Diseases (ICD) 27 codes for diagnoses and Logical Observation Identifiers Names and Codes (LOINC) 28 for laboratory values. Structured data that are not part of UMLS ontologies but are a standard component of the Epic data model, such as Social Vulnerability Index and social drivers of health, are mapped to Epic-released values used to facilitate a within-vender interoperability ontology. For many organizations, much of this mapping is already completed to support clinical workflows. 25 Non-discrete and custom elements are mapped to the best match from these code sets. When no matching discrete value exists in a code set, data are mapped using free text, which automatically maps for exact string matches. Values that cannot be matched are not exposed to users. The frequency of unexposed data varies by data type, but is rare due to mapping thresholds required to data. Data are submitted on an ongoing, near-real-time basis from contributing organizations ( Fig. 1 ). Patient matches made at participating organizations through Care Everywhere, Epic's within-vendor interoperability network, are sent as cryptographically hashed IDs, allowing chart deduplication without introducing re-identification risk. 25 After an organization begins submitting, historical data are backloaded to the date the organization began using its Epic EHR. When data elements are added or updated, queries are sent to trigger targeted data submissions without required manual intervention.

#### Data Quality

Prior to exposing contributed data in Cosmos, groups work with Epic to validate submitted data quality by comparing metrics for their organization to community-wide standards for organizations of the same type (e.g., outpatient-only clinics are exempt from inpatient metrics). Local regulations preventing contribution of certain data—such as HIV/AIDS 29 —are accounted for. Metrics follow Kahn's Harmonized Data Quality Assessment Framework of Completeness, Conformance, and Plausibility. 30 Thresholds for each metric are defined based on the completeness level expected to be needed to draw accurate conclusions, conformance, and plausibility evaluation by clinicians and data scientists, and comparisons with published references. Table 1 demonstrates a subset of metrics and associated thresholds for hospital admissions. As data elements are added, new metrics are published. Organizations correct submissions not meeting thresholds by updating mappings or system configuration. The vendor also monitors implementing organizations' submission volumes to identify deviations from local systems' volumes and take corrective action as needed. Once thresholds are met for all applicable elements, the organization's data are made available for use. To maintain quality over time, metrics and contribution volume are regularly reassessed to date on a quarterly basis.

**Table 1:** Subset of data quality metrics released with hospital admissions

Completeness	Conformance	Plausibility
Has patient link (>95%) Has encounter link (>95%) Has encounter type (>95%) Has admission instant (>95%) Has discharge instant (>95%)	Encounter type does not indicate hospitalization (<1%) Has discharge disposition of deceased but no death date (<1%) Discrepancy between documented coverage and payment flags (<1%)	Discharge instant occurs before the patient's birth or admission instant occurs after patient's death (<1%) Length of stay longer than a year (<1%) Discharge date occurs after next admission (<1%)

Note: Associated thresholds included in parentheses.

Users with access to Cosmos' web portal can see data quality metrics for the entire dataset and their own organization. Each metric displays the Structured Query Language (SQL) code used for its calculation, promoting transparency serving as a starting point for cohort analysis. Users can benchmark their organization against the Cosmos community to self-identify opportunities to improve their organization's data quality.

#### Available Datasets

There are two versions of the Cosmos dataset, differentiated by type of anonymization and access. Both datasets are static snapshots that, to date, have typically been updated every 2 weeks. The first meets the HIPAA definition of a limited dataset (LDS), 14 15 with direct identifiers removed in local systems prior to data transmission. As previously described, users may only interact with summary level visualizations of this dataset ensuring a de-identified view of data. 25 The second is derived from the LDS with additional anonymization meeting HIPAA's expert determination de-identification (EDDI) standard. 17

As part of the expert determination process, a third-party data advisory service creates an independent Re-identification Risk Determination report for the EDDI dataset on a quarterly basis. As per industry standard, this report outlines the transformations and access controls needed to minimize re-identification risk while preserving research utility. While the LDS contains exact dates, each patient in the de-identified dataset receives a consistent, randomly assigned date shift forward, which preserves the sequence and intervals of events. While postal codes are available in the LDS, geographic granularity is limited to U.S. state in the de-identified dataset. Geographically defined indices are attached at the patient level in both datasets based on patients' current residence. Some values are obfuscated with Gaussian noise in the de-identified dataset to further reduce re-identification risk. 31 32

#### International Contributors

All data reside in a single environment hosted in the U.S. All elements in the dataset are submitted by all participating organizations regardless of locale, provided they are documented and submission is permitted in local regulations. Each participating group maintains responsibility for ensuring compliance with local laws and regulatory frameworks prior to data submission. Organizations submit data using the code sets in which they are documented. When possible, data are mapped on the Cosmos host to international standards, such as LOINC or SNOMED. In cases where standards vary by region, data are presented with their corresponding code type. When no regional standard exists, data are sent as free text and string-matched to a standard code set when possible. Locale-specific differences in demographics, geography, or workflows may drive exceptions during the data-quality evaluation. Both datasets indicate country of origin, allowing users to filter cohorts.

#### Analytic Tooling

Users access Cosmos through a web portal containing distinct tools for each database which differ in access requirements and analytic tooling ( Table 2 ). All users can interact with the de-identified view of the LDS through an Epic-developed no-code visualization and analysis application called SlicerDicer. This tool generates summary statistics, reports values 1 to 10 as “10 or fewer,” and does not permit access to line-level data to prevent patient reidentification. Users with required paid training can use the Data Science Virtual Machines (DSVMs) to access the de-identified database at a line-level granularity for projects requiring customizable analyses. Both tools support collaborating on queries, saving resources for longitudinal analysis, and exporting aggregate results. As queries may be computationally intensive, a pseudorandom sample (currently approximately 1% of the overall data) of each database is provided with each snapshot to permit preliminary investigations with the respective toolset.

**Table 2:** Comparison of Cosmos analytic toolsets

Feature	No-code data visualization and analysis tool	Data science virtual machines
Access requirements	All users of Cosmos web portal	Requires training on Cosmos data models, data literacy, and responsible security practices
Underlying dataset	Limited data set	Expert-determined de-identified database
Data granularity	Aggregate analyses Counts less than 10 masked	Line-level access to data
			Data availability	Granular geographic location data, such as county and ZIP code Specific dates	Limited to state- and country-level analysis Date shifting
Analytic tooling	Epic-developed application Graphical user interface using system-defined data models a and filters and custom-built filters composed of system filters Descriptive statistics (counts, minimum, maximum, mean, percentage, percentile, standard deviation, confidence interval) Risk and odds ratios Sequential event tracking and outcome event analysis Unmatched cohort studies	Third-party industry standard tools for Structured Query Language (SQL), Python, R, and code management. More than 750 software packages to support their use Users may request additional packages Allows for custom analysis and modeling using line-level data
			Sample	Pseudorandom sample Demographics statistically similar to full dataset Automated asynchronous processing on full dataset	Pseudorandom sample Demographics display significant differences from full dataset Manual submission of queries to full database
						Results export	Custom metric calculation available within interface Aggregate counts may be exported for local analysis	File review process required to transfer resources in or out Aggregate counts may be exported for local analysis (line-level export prohibited)
									Collaboration	Sessions can be shared with other users (individually or to all Cosmos portal users)	Project team collaboration through shared SQL databases, network filestores, and code management repositories Code management repository accessible to users outside of project context for open-access sharing

a System-defined database structures that organize related data elements representing specific clinical entities (e.g., patients, encounters, etc.).

#### User Access and Support

Participation in Cosmos continues to be an opt-in service for Epic EHR customers. 25 The platform is supported by the Cosmos Governing Council (CGC), a body of clinicians, researchers, and informaticists from participating healthcare organizations. Governing council members are nominated for election by the participating community and voted on by one designated representative at each participating organization. This body supports the integrity of the platform by enforcing the framework for member organization participation and responsible use and privacy practices, described in a policy document titled the “Cosmos Rules of the Road” (CROTR).

Access is granted in accordance with the CROTR. Before obtaining access, users must be an employee of a submitting organization or affiliated institution (e.g., associated university), have analytics access within their organization's EHR system, and acknowledge an agreement for responsible data use and HIPAA best practices for patient privacy protections. For each project, users must specify conflicts of interest, including funding sources, before access is granted. Query and session auditing allow the CGC and Epic to enforce this agreement according to procedures specified in the CROTR.

Most work undertaken in Cosmos is unfunded. Projects with external funding require approval from both Epic and the CGC to ensure alignment with the goals of Cosmos as stated in the CROTR. Fees may be charged when funding is received or additional resources are needed. Because data are accessed in a manner that prevents re-identification of patients, Epic and the CGC do not require Institutional Review Board (IRB) approval for using either dataset, though member organizations are responsible for their own IRB policies. 33 At the time of submission, several have deemed Cosmos as non-human subjects research and have standing exemptions or templated language to support Cosmos projects.

Users receive support through their organization's dedicated structure, supported by a designated Epic contact, a portal-based catalogue describing data relationships, and web-based repository that houses documentation describing data quality and best practices. Collaboration is supported through project sharing, open-source code-sharing within the DSVMs, and vendor-facilitated user group meetings.

### Descriptive Analysis

#### Descriptive Statistics

Data for descriptive analyses, including data in the Results section and all figures and tables were obtained from the Cosmos LDS April 24, 2025 database snapshot, except where otherwise noted. Proportions were compared with Chi-square testing where applicable. Analyses were performed in Microsoft Excel 365. Figures were generated in R (version 4.5.0) with ggplot2. 34 35

#### Review of Funded Studies and Publications

A list of peer-reviewed publications using Cosmos is available via the public web site 36 and approved funded studies known to the vendor are accessible within the web portal. The CGC has approved studies with external funding sources, including National Institutes of Health (NIH), Centers for Disease Control and Prevention (CDC), pharmaceutical manufacturers, and not-for-profit organizations. To date, over 140 peer-reviewed manuscripts utilizing Cosmos have been published. The authors used this list to perform a non-systematic literature review highlighting data elements within Cosmos that have demonstrated potential to enable novel research.

## Results

### Growth of Cosmos

**Fig. 2:**
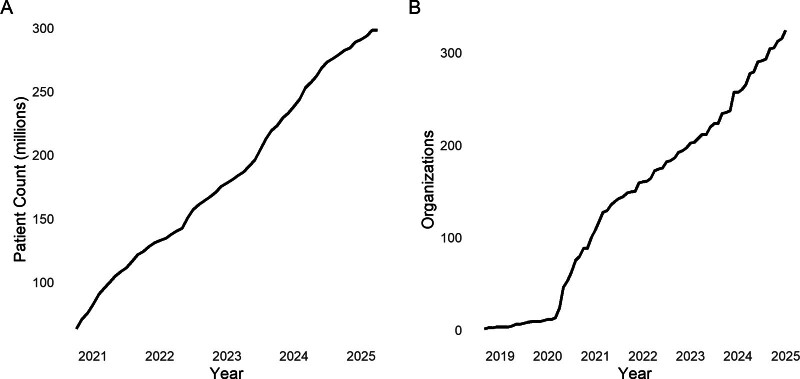
Cosmos characteristics as of May 2025. ( **A** ) Count of unique patients with data present in Cosmos, 2021–2025. ( **B** ) Total of unique organizations submitting data to Cosmos over time, beginning August 1, 2018, with the first submitting organization.

**Fig. 3:**
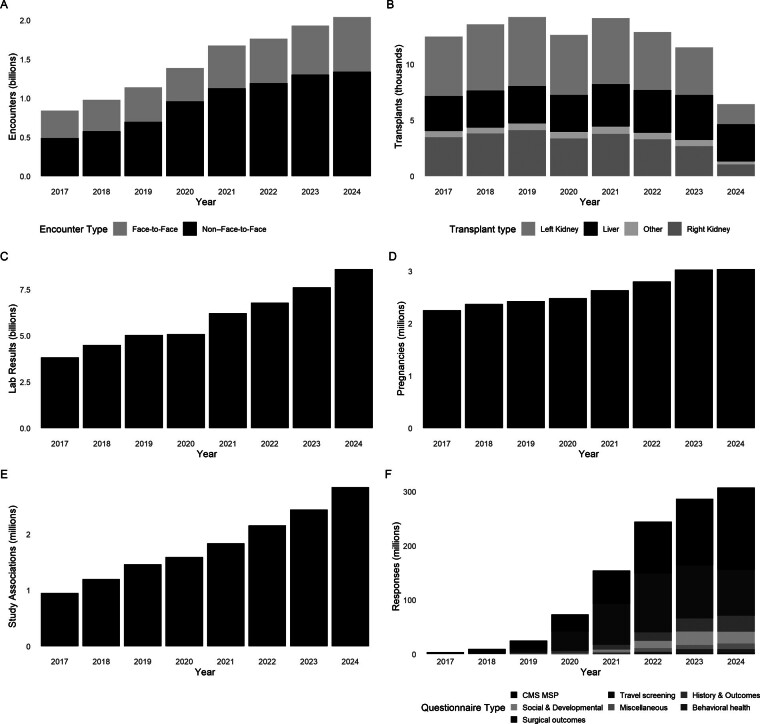
Volume of data types by year. ( **A** ) Count of encounters occurring each year, separated by face-to-face and non–face-to-face. ( **B** ) Count of organ transplants occurring each year, separated by organ. ( **C** ) Count of laboratory component results each year. ( **D** ) Count of pregnancies occurring each year. ( **E** ) Count of patient research study associations occurring each year. ( **F** ) Count of patient-reported outcomes recorded each year separated by category. All data range from 2017 to 2024. Data definitions provided in Supplementary Table S1 (available in the online version only).

Ongoing addition of data elements and participating organizations has resulted in consistent year-over-year growth: as of April 2025, data exist for 300 million deduplicated patient records ( Fig. 2A ) and 337 organizations ( Fig. 2B ). Volumes of data elements including encounters, laboratories, pregnancies, research study associations, transplants, and patient reported outcomes have generally doubled or more than doubled between January 2017 and December 2024 ( Fig. 3 ).

### Depth of Data

**Fig. 4:**
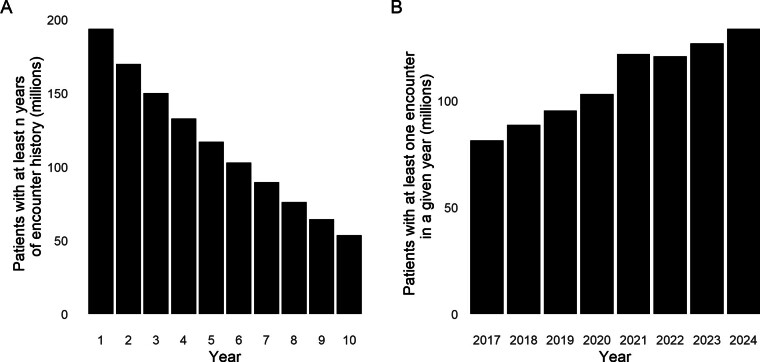
Encounter densities. ( **A** ) Counts of patients with at least *n* number of years of encounters in Cosmos. Values were calculated by taking the difference between each patient's first encounter's start date and the last encounter's end date. ( **B** ) Counts of patients with at least one encounter in a given year as a function of time.

**Fig. 5:**
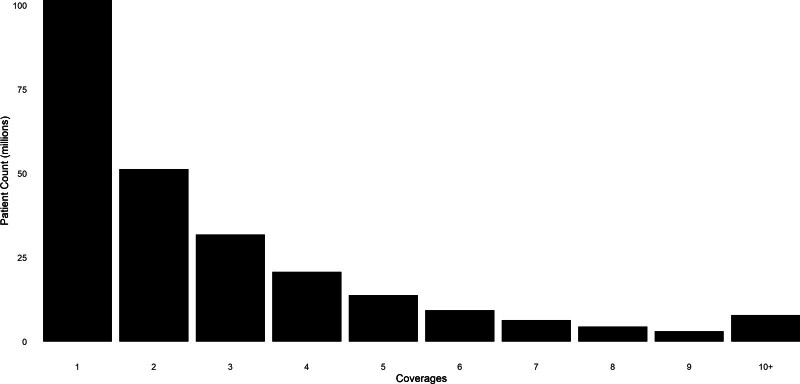
Number of patients by distinct coverage. Number of patients as a function of the number of distinct coverages present in Cosmos.

At the time of publication, 63% (118 million) of patient records have information contributed by only one care organization, 25% (74 million) from two, 9% (27 million) from three, 3% (8 million) from four, and 1% (3 million) from five or more organizations. A total of 117 million patients (39.03% of the Cosmos population) have 5 or more years of data, and 53 million patients (17.84%) have 10 or more years of data ( Fig. 4 ). The longest patient records extend 28 years; 39.8 million patients (13.28%) have encounters billed to 5 to 10 distinct insurance coverages, while an additional 5.8 million patients (1.95%) have encounters billed to more than 10 coverages ( Fig. 5 ).

### Representativeness

Patients in Cosmos from the U.S. display a distribution similar to the 2020 U.S. Census across categories such as race, 37 ethnicity, 37 sex, 38 Social Vulnerability Index, 39 age, 38 and primary coverage 40 41 42 ( Table 3 ). Across the same categories, pseudorandom samples for the LDS ( Table 4 ) and EDDI ( Table 5 ) display a similar pattern of distribution to their corresponding complete dataset, though statistically significant differences were seen between the sample and complete EDDI databases. Published studies suggest utility of Cosmos data in evaluating common and rare conditions including diabetes, 43 trisomy-21, 44 autoimmune lymphoproliferative syndrome, 45 and Glanzmann thrombasthenia. 46

**Table 3:** Comparison of Cosmos de-identified dataset and U.S. Census

Attribute	Category	Prevalence (Cosmos U.S. patients) *N* = 298,057,109	Prevalence (census) *N* = 334,914,896
Race a	White	73.11%	75.44%
	Black or African American	16.29%	16.38%
	Asian	4.85%	8.56%
	Native Hawaiian or Other Pacific Islander	0.59%	0.49%
	American Indian or Alaska Native	1.04%	2.48%
	Other	12.64%	2.18%
Legal sex	Female	52.48%	50.76%
	Male	47.52%	49.24%
Ethnicity	Hispanic or Latino	15.64%	18.73%
	Not Hispanic or Latino	84.36%	81.27%
Social Vulnerability Index (percentile)	0–25	27.69%	25.15%
	25–50	25.72%	25.17%
	50–75	23.99%	24.72%
	75–100	22.60%	24.96%
Age in years	0–15	13.69%	18.60%
	15–30	17.21%	20.29%
	30–45	20.60%	19.47%
	45–60	17.24%	19.39%
	60–75	17.57%	15.58%
	75+	13.69%	6.68%
Primary or secondary health insurance financial class	Self-pay	8.35%	9.11%
	Medicare	15.70%	18.37%
	Medicaid	20.34%	20.94%
	Private/Other	85.59%	73.39%

Notes: Prevalence of individuals in Cosmos (U.S. only) and the U.S. Census with selected responses for race, legal sex, ethnicity, Social Vulnerability Index, age, and financial class of primary or secondary insurance coverage. Individuals without documented responses are excluded from calculation and proportions in the corresponding category. Data from April 24, 2025, database snapshot.

a Includes any race documented for individual (e.g., primary, secondary, etc.) up to six documented races.

**Table 4:** Example demographic comparison of pseudorandom sample and full limited data set

Attribute	Category	Prevalence (sample) *N* = 2,983,028	Prevalence (complete) *N* = 298,642,708	*P* -value
**Race**	White	61.93%	61.95%	0.56
	Black or African American	13.81%	13.80%	
	Asian	4.10%	4.12%	
	Native Hawaiian or Other Pacific Islander	0.50%	0.50%	
	American Indian or Alaska Native	0.88%	0.88%	
	Other	10.74%	10.71%	
	Not documented	14.70%	14.70%	
**Legal sex**	Female	52.35%	52.40%	
	Male	47.50%	47.45%	
	Other	0.14%	0.14%	
	Not documented	0.01%	0.02%	
**Ethnicity**	Hispanic or Latino	12.51%	12.48%	0.33
	Not Hispanic or Latino	67.17%	67.20%	
	Not documented	20.32%	20.32%	
**Social Vulnerability Index (percentile)**	0–25	13.03%	13.00%	0.60
	25–50	12.03%	12.04%	
	50–75	11.19%	11.22%	
	75–100	10.51%	10.51%	
	Not documented	53.24%	53.24%	
**Age in years**	0–15	13.63%	13.64%	0.06
	15–30	18.19%	18.17%	
	30–45	20.54%	20.55%	
	45–60	17.14%	17.18%	
	60–75	17.47%	17.41%	
	75+	13.03%	13.04%	
	Not documented	∼0.00%	∼0.00%	
**Primary or secondary health insurance financial class**	Self-pay	4.73%	4.74%	0.82
	Medicare	8.88%	8.89%	
	Medicaid	11.54%	11.53%	
	Private/Other	48.43%	48.46%	
	Not documented	43.00%	43.00%	

Note: No significant differences exist in distributions of demographic attributes between datasets in the May 7, 2025 snapshot, though similarity of distribution for each selected attribute varies from snapshot to snapshot. Undocumented values were included in analysis to reflect no bias away from patients with incomplete documentation. *P* -values calculated by Chi-square.

**Table 5:** Example demographic comparison of pseudorandom sample and complete de-identified dataset

Attribute	Category	Prevalence (sample) *N* = 2,979,487	Prevalence (complete) *N* = 298,607,898	*p* -Value
Race	White	61.91%	61.95%	1.3 × 10 ^−2^
	Black or African American	13.75%	13.80%	
	Asian	4.08%	4.12%	
	Native Hawaiian or Other Pacific Islander	0.50%	0.50%	
	American Indian or Alaska Native	0.88%	0.88%	
	Other	10.72%	10.71%	
	Not documented	14.70%	14.70%	
Legal sex	Female	52.42%	52.40%	0.21
	Male	47.43%	47.45%	
	Other	0.03%	0.03%	
	Not documented	0.13%	0.13%	
Ethnicity	Hispanic or Latino	12.51%	12.48%	0.20
	Not Hispanic or Latino	67.18%	67.20%	
	Not documented	20.31%	20.32%	
Social Vulnerability Index (percentile)	0–25	12.86%	12.89%	0.02
	25–50	12.06%	12.06%	
	50–75	11.24%	11.26%	
	75–100	10.51%	10.55%	
	Not documented	53.33%	53.24%	
Age in years	0–15	13.51%	13.67%	6.5 × 10 ^−14^
	15–30	17.36%	17.28%	
	30–45	20.61%	20.61%	
	45–60	17.29%	17.24%	
	60–75	17.57%	17.55%	
	75+	13.66%	13.64%	
	Not documented	0%	0%	
Primary or secondary health insurance financial class	Self-pay	4.71%	4.68%	1.7 × 10 ^−10^
	Medicare	8.85%	8.87%	
	Medicaid	11.42%	11.48%	
	Private/Other	48.00%	48.16%	
	Not documented	43.47%	43.30%	

Note: In data from May 7, 2025, snapshot, a significant difference in distributions is observed in the sample and the complete dataset for several selected demographic attributes. Undocumented values were included in analysis to reflect no bias away from patients with incomplete documentation. *P* -values calculated by Chi-square.

### Notable Data Elements

#### Perinatal Health

Deterministic mother–child EHR linkages in Cosmos yields longitudinal data that have been used to explore the impact of prenatal maternal health conditions on birth outcomes 23 and delivery encounter vitals on maternal readmission risk. 47 The platform may enable investigation of associations between prenatal prescriptions and children's health 48 49 and correlations in outcomes among siblings. 50

#### Transplants

Comparative analysis shows near parity between demographic and clinical characteristics of transplant episodes in Cosmos and the Scientific Registry of Transplant Recipients database, suggesting that the platform might be promising for scalable transplant management research. 51 52 Comprehensive data before and after transplantation may enable investigations of longitudinal outcomes within detailed cohorts.

#### Insurance Coverage Payers and Financial Class

Cosmos includes billing data at the encounter level, categorized by financial class including Medicare, Medicaid, Medicare Advantage, self-pay, and private coverage with primary coverage denoted for patients with multiple active coverages. This data may enable comparisons across insurance types and investigations into the impact of policy decisions on care utilization. 53 54

#### Research Study Association

Cosmos contains information on patients' associations with research studies, including the study type and timeframe of participation, allowing researchers to assess trends in study participation. 55 To protect patient and organization privacy, identifiable details of specific studies are excluded.

## Discussion

The Cosmos platform demonstrates the use of a standards-based information exchange architecture to aggregate EHR data across diverse health systems forming a within-vendor repository. Unique in its volume and depth of data, the database enables longitudinal observational research across a growing spectrum of domains including perinatal health, transplants, insurance coverage, and participation in research trials. The following section discusses specific strengths and limitations relative to other existing data sources and opportunities for future work.

Similar to other multi-institutional health record databases, analyses in Cosmos frequently exhibit high statistical power due to large sample sizes but are susceptible to the big data paradox, wherein highly significant results might not reflect clinically meaningful discoveries. 11 This abundance of statistically significant results increases the risk of false discoveries. For example, a researcher might observe a statistically significant rise in a specific comorbidity, which in reality may reflect a shift in billing practices rather than a true change in clinical pathology. As the number of healthcare organizations participating in Cosmos and the volume of encounters continue to grow, the importance of distinguishing documentation variances from biological trends intensifies. As such, researchers should account for biases EHR data are subject to, including collider, selection, information, and sampling bias. 11

While there is a growing availability of data elements, Cosmos lacks several, which may restrict study feasibility. Foremost is the reliance on structured data as unstructured and non-discrete data elements are not available. Extracting insights from clinical notes, images, and genomic sequencing might expand the potential of Cosmos and further differentiate the platform from other healthcare data sources. 56 As new data elements are added and sample sizes increase, ongoing implementation must follow best practices to account for increases in risk of individual reidentifiability that may arise. 57 58 59 60

Researchers should also note that transforms required for the de-identified dataset may make the LDS better suited for some studies. For example, works requiring exact dates may not be feasible in the de-identified dataset due to date-shifting, and geographic insights may be limited to the state or country level. In these cases, analysis can be performed using the data visualization tool which provides less customizability relative to the flexibility of the data science tools, which are more time intensive and requires training.

While necessary to minimize re-identification risk, the design of the platform inherently limits the use of certain data. Notably, line-level data from the de-identified dataset cannot be connected to external line-level data that are not already associated at the patient level in Cosmos. Researchers should evaluate whether existing elements can approximate these measures or consider combining aggregate Cosmos data with reference datasets. Likewise, patients cannot be re-identified for clinical validation purposes, unlike work performed using single-institutional and certain limited datasets.

Initial analysis suggests Cosmos may be able to complement specialty-specific data repositories, 51 52 and our findings demonstrate a U.S. population in Cosmos that aligns with the most recent U.S. Census data. Additional analysis is recommended to understand the representativeness of specific disease burden across a broader spectrum of conditions within Cosmos and assess the generalizability of new data types to facilitate their adoption. No studies have yet been published validating data for genetic variants, cancer staging, or patient-reported outcomes, though their use might permit extensions of previous uses of Cosmos. 51 52 61 62 63 64 Foundational analyses should document best practices for data cleaning, benchmark against established datasets, and evaluate strengths and limitations of Cosmos within the domain.

Cosmos currently receives data from four countries. As Epic EHR systems are currently deployed in 16 countries, there is opportunity for organizations in additional locales to join and position Cosmos as an asset for investigating national differences in medical trends. In addition to locale-specific legal and jurisdictional implications, 65 66 international contribution to Cosmos introduces three key considerations for researchers. First, researchers might want to account for the country in which care was provided, though the appropriate methods may depend on the goals of analysis. Second, comparative analyses across nations must address sample size biases; while Cosmos receives submissions from more than 330 U.S. organizations, non-U.S. sources may represent a more limited subset of their national populations. Third, different code systems, chart completeness, and data collection practices across nations must be accounted for. Further foundational analysis is required to determine best practices for incorporating data across countries to complete effective comparative research.

Cosmos' data backload extends the longitudinality of patient records before the date a health system began contributing data to Cosmos. The ability to deduplicate patient charts across organizations and insurance coverages without introducing a risk of reidentification differentiates the platform from similar databases. However, it is worth noting that deduplication efforts in Cosmos are deterministic, relying on documented interoperability and deduplication events within a patient chart, which suggests non-deduplicated patients as a potential source of error on measures performed on Cosmos data. Nevertheless, these features enable detailed sequential event analyses across multiple healthcare organizations, allowing researchers to investigate long-term effects of treatments and diseases—sometimes several decades for patients with EHRs at organizations who implemented an Epic EHR decades ago. Studies spanning multiple decades might need to account for the possibility of bias introduced by relying on data from limited sources.

**Fig. 6:**
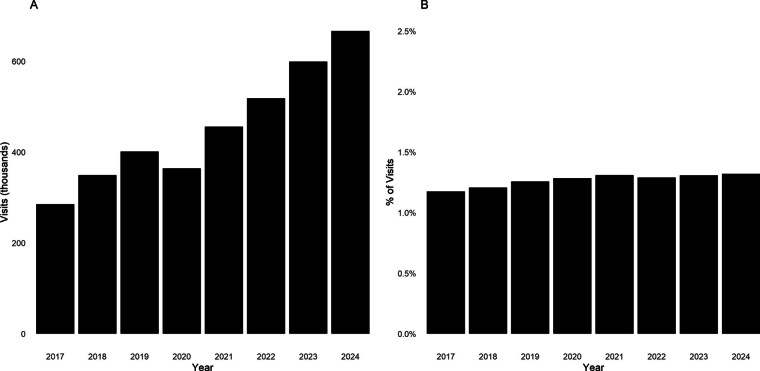
Emergency department visits for wrist-related billed procedure. ( **A** ) Count of emergency department visits with a wrist-related diagnosis as a function of time. ( **B** ) Percentage of emergency department visits with a wrist-related diagnosis as a function of time.

The nature of the platform's growth necessitates reporting rates rather than counts to adjust for growth over time ( Fig. 6 ). Additionally, some elements show disproportionately high growth due to adoption of workflows with discrete documentation. This can be accounted for by normalizing rates to the subset of sites actively using the discrete fields or restricting analysis to periods after documentation practices stabilize.

While historical data enables longitudinal analysis, the recency of data allows Cosmos to serve as a timely source for conducting research informing policy 67 68 69 70 71 72 73 74 75 and public health surveillance and initiatives, particularly in the context of the United States. 76 As with any recent real-world data, researchers must be mindful of workflow realities, such as pending results, that may amend documentation and affect conclusions. To mitigate the inclusion of incomplete information, data are held from the submission queue in the days preceding the creation of a database snapshot. Consequently, recent data might reflect a reduced volume in the days leading up to a snapshot. Though this might limit use cases necessitating real-time data, researchers may consider restricting queries to the last date of complete submission as documented in the web portal.

User engagement has seen consistent growth since 2021, though it has not been uniform across healthcare organization types and opportunities remain for cross-organization collaboration. 76 At the time of manuscript submission, 5,143 active users from 292 participating organizations, including 1,081 data science users from 168 organizations have access to Cosmos. 77

While pseudorandom sampling aims to permit proof-of-concept generation with lower computing costs, differences between pseudorandom samples and corresponding complete datasets justify a need for computational infrastructure to conduct final analyses. The enhanced analytic infrastructure—specifically data science tooling using the de-identified data—has facilitated multifactorial epidemiological studies 78 79 and detailed descriptive statistics, 80 81 82 and the development of predictive models, 83 84 and early research indicates Cosmos may be capable of producing novel insights with generative AI technology. 85 However, resource constraints to date have limited some computationally intensive tasks that could become possible over time with the addition of computational resources.

Cosmos also supports applications within local EHR systems and for multi-center prospective clinical trial site recruitment and patient identification that leverage insights derived from its data. Tools such as clinical decision support or information displays to assist in patient care, such as specialized grow charts and community antibiograms, are being developed and evaluated within the web portal and are being integrated into local EHR systems. 86 87 For multi-center clinical trials, users can identify eligible patient criteria across all patients in Cosmos and then send pointers of eligible patients to local EHR systems to facilitate initial trial site recruitment and then eventually patient recruitment. 88 However, operationalization of these applications may encounter challenges in generalizability across diverse health systems and data governance policies. These barriers underscore the need for validation, local adaptation, and cross-institutional coordination before deploying such applications.

The data quality infrastructure behind Cosmos focuses on transparency, allowing researchers to identify data fitness considerations early in their investigation, including what types of analysis are possible and what data cleaning is necessary. Despite these strengths, limitations inherent to within-vendor, real-world EHR data remain. Completeness of elements which are only documented for a subset of the population—such as social drivers of health—may introduce bias into analyses. Additionally, despite vendor-level standardization, variability in documentation practices, workflows, and coding across health systems can affect consistency. Because researchers must work with information as originally recorded, these factors underscore the role of independent data quality assessments in parallel with the vendor's validation processes. When identified, users may choose to exclude sources contributing incomplete or non-conforming data.

Finally, Cosmos reflects care documented within Epic EHR systems. Factors influencing patient engagement with health systems can result in certain groups being underrepresented relative to the overall population. 89 Accordingly, researchers must also evaluate representativeness for populations of interest. Opportunities remain to establish identification algorithms for current conditions or other patient characteristics based on evidence from the EHR. Establishing common standards is a prerequisite to understanding and comparing representativeness across data sources. Additionally, data not typically documented in participating health systems' records, such as out-of-hospital mortality and prescription adherence, may present challenges for studies' feasibility. Despite these challenges, Cosmos distinguishes itself from other real-world data sources, such as claims databases, specialty registries, or single-organization databases, through its scale and longitudinality of patient records, the breadth of clinical and non-clinical elements present, and the recency of available data.

## Conclusion

The increasing use of Cosmos underscores an industry-wide trend toward tools that enable large-scale healthcare research within the evolving landscape of healthcare delivery. As the number of organizations participating in Cosmos expands and data elements are added, the research community has a continued opportunity to demonstrate the platform's feasibility for generating medical knowledge and building upon uses of Cosmos for observational research, disease surveillance, and clinical application of insights.

## Clinical Relevance Statement

Seamless data aggregation between healthcare systems and population-level analytics may be an important step in the evolution of evidence-based medicine and large-data medical research. This paper demonstrates the growth of Cosmos since its inception, highlighting the platform's strengths and weaknesses including areas where researchers may find opportunity for unique insights.

## Contributors' Statement

S.K.M.: conceptualization, data curation, formal analysis, investigation, methodology, project administration, software, visualization, writing—original draft, writing—review and editing; M.H.: conceptualization, data curation, formal analysis, investigation, methodology, software, visualization, writing—original draft, writing—review and editing; Y.T.: conceptualization, funding acquisition, writing—review and editing; D.C.K.: conceptualization, funding acquisition, supervision, validation, writing—review and editing; S.S.C.: conceptualization, writing—review and editing; H.F.: conceptualization, methodology, supervision, writing—original draft, writing—review and editing.
