# Supplementary material for: Advancing Real-World Evidence Generation: Growth and Lessons Learned from the Early Years of Cosmos
Source: ACI open. 2026 Apr 24;10(1):e10–23. doi: 10.1055/a-2842-3407 (PMC13404524; doi:10.1055/a-2842-3407)
Supplement: Supplementary file 1 — Supplementary Material [file 10-1055-a-2842-3407_28552730.pdf]

Supplementary Table 1. Data definitions

Term	Definition
**Face-to-face encounters**	An encounter with a type of Allied health, Anesthesia, Anesthesia event, Ancillary procedure, Anticoagulation visit, Appointment, Assisted living/skilled nursing, Audiology, Behavioral health, Case management, Clinical support, Confidential, Diagnostic services, Education, Emergency, Emergency to inpatient, External hospital admission, Fetal care consult, Fetal procedure, Follow-up, Genetics,Group Visit, Home care visit, Hospice F2F visit, Hospice admission, Hospice physician oversight, Hospital, Hospital Outpatient Visit, Hospital outpatient visit to inpatient, Immunization, Induction, Infusion, Initial prenatal, Injection, Inpatient admission, Lactation consult, Lactation encounter, Multidisciplinary visit, NST, Nurse only, Nursing home, Nutrition, Occupational/Physical therapy, Office visit, Oncology survivorship, Ophth exam, Other hospital encounters, Postpartum visit, Procedural consult, Procedure visit, Radiology appointment, REI, Research encounter, Routine prenatal, Sleep study, Social work, Speech therapy, Surgery, Surgical consult, Telemedicine, Transplant evaluation, Transplant follow up, Treatment, Urgent care, Walk-in, Well child
**Non-face-to-face encounters**	An encounter with a type of Abstract, Admission orders, Ancillary orders, Billing encounter, Biometric visit, Cardiology conference, Clerical orders, Clinical documentation only, Committee review, Community care management, Community orders, Consent form, Consult, COVID testing, Device check, Documentation, E-Consult, E-Consult community order, Employee health, Enrollment, EpicOnHand encounter, E-Prescribe, Evaluation, E-Visit, External communication, External contact, History, Home care update, Home infusion, Home monitoring, Immunotherapy, Intake, Lab, Lab requisition, Legacy encounter, Letter (out), Long term care, Medication management, Meds only (web), Miscellaneous, Mobile order only, MyChart, Nurse triage, Orders only, Outside procedure, Patient care review, Patient message, Patient outreach, Patient self-triage, Patient web update, Pharmacy visit, Plan of care documentation, Post mortem documentation, Pre-Admission testing, Pre-Evaluation, Pre-op/Pre-procedure orders, Procedure pass, Questionnaire series submission, Reconciled outside data, Recurring plan, Referral, Referral triage, Refill, Registration, Release of information, Results follow-up, Results only, Rx refill authorize, Scanned document, Social care application, Specialty pharmacy, Telephone, Telephone visit, Transcribe orders, Travel, Tumor board conference, Update
**Lab results**	All lab component result in Cosmos.

**Research study associations**	All active research study associations in Cosmos in a given year.
**Pregnancy episodes**	All pregnancy episodes in Cosmos. Dates are estimated from the delivery date based on an estimated pregnancy span. Pregnancies are counted in the year they began.
**Transplants by organ**	**Left kidney**<br>**Liver:** Includes liver, right liver lobe (segs 5, 6, 7, 8) without middle hepatic vein, right liver lobe, left lateral liver, left liver lobe, left lateral liver (segs 2, 3), right liver lobe (segs 5, 6, 7, 8) with middle hepatic vein, trisegmental liver (segs 1, 4, 5, 6, 7, 8), trisegmental liver, left liver lobe (seg 1, 2, 3, 4), and left liver lobe (segs 2, 3, 4)<br>**Right kidney**<br>**Miscellaneous**: Includes kidneys en block; kidney, side unknown; intestines, small intestine, heart, segmental pancreas
**Patient-entered questionnaire responses by category**	Additional questionnaires are mapped and brought into Cosmos over time, prioritized by relevance to common research questions or researcher requests. Only a patient's most recent response is recorded. At the time of publication, there are 416 unique screeners in Cosmos, broken down into the following categories for comprehensibility:<br>**Travel Screening**<br>**CMS Medicare Secondary Payer (MSP)**<br>**Social & Developmental:** Includes Health Related Social Needs (HRSN) screenings and Survey of Well-Being of Young Children (SWYC) screenings<br>**Behavioral Health:** Includes AUDIT-C, PHQ-9, GAD-7, Edinburgh, and C-SSRS<br>**Surgical Outcomes**: Includes HOOS/KOOS and THA/TKA<br>**History & Outcomes:** Includes PROMIS and patient-entered histories<br>**Miscellaneous:** Includes COVID-19 screenings, telehealth questionnaires, and other clinical screenings
